# Use of a self-assembling peptide to control complications associated with endoscopic balloon dilation of refractory anastomotic stricture

**DOI:** 10.1055/a-2277-5968

**Published:** 2024-03-14

**Authors:** Takehide Fukuchi, Shigeru Iwase, Kingo Hirasawa, Shin Maeda

**Affiliations:** 1 36993 Department of Gastroenterology, Fujisawa City Hospital, Fujisawa, Japan; 2 26437 Division of Endoscopy, Yokohama City University Medical Center, Fujisawa, Japan; 3 26438 Department of Gastroenterology, Yokohama City University School of Medicine Graduate School of Medicine, Yokohama, Japan

PuraStat (3-D Matrix, Tokyo, Japan) is a novel self-assembling peptide hydrogel developed as a hemostatic agent that is believed to be effective in reducing the risk of delayed perforation due to excessive cautery burns 1 2 3 . Additionally, studies in animal models have suggested the potential of PuraStat for preventing esophageal strictures after endoscopic submucosal dissection for esophageal cancer 4 5 . In this report, we present a case in which the self-assembling peptide gel effectively managed esophageal stricture and safely addressed complications related to endoscopic balloon dilation (EBD) ( Video 1 ).

A self-assembling peptide hydrogel is used to control complications associated with endoscopic balloon dilation of refractory anastomotic stricture.Video 1

The patient was a 71-year-old man who developed a severe anastomotic stricture after esophagogastrostomy for esophageal cancer. EBD for the stenosis was performed repeatedly every 2 months after surgery, but no improvement was observed ( Fig. 1 **a** ).

**Fig. 1:**
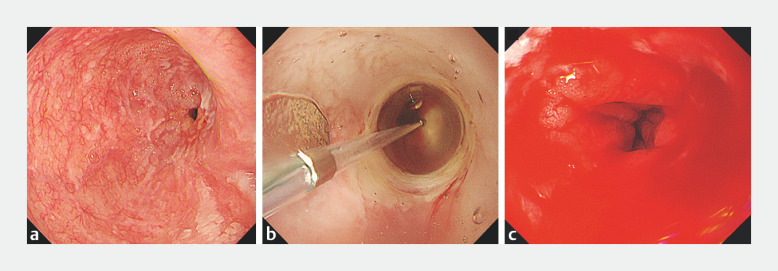
**a** Anastomotic stricture following esophagogastrostomy. **b** The stricture was dilated to 12 mm (8 atm) using EBD. **c** Identification of the bleeding source was challenging.

The 23rd EBD was performed to 12 mm (8 atm) using multistage dilation balloons (Micro-Tech, Nanjing, China) as usual ( Fig. 1 **b** ). Immediately after deflation, active bleeding was observed. The bleeding source was on the posterior wall and submerged due to gravity which made it difficult to identify; the wound appeared to be deep ( Fig. 1 **c** ). Given the complexity and risk of alternative hemostasis methods, we applied PuraStat achieving hemostasis in approximately 1 minute ( Fig. 2 ).

**Fig. 2:**
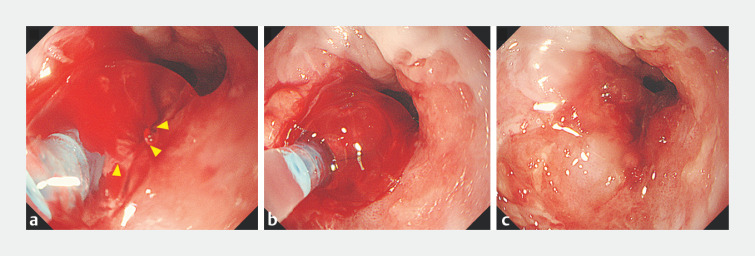
**a** Application of PuraStat self-assembling peptide hydrogel allows simultaneous visualization and hemostasis (arrows). **b** Application of pressure at the bleeding site to create a bulge. **c** Complete cessation of bleeding within approximately 1 minute.

Despite the persistent and challenging nature of the stricture, it gradually improved, and the frequency of EBD procedures decreased significantly after the introduction of PuraStat. The use of PuraStat as a wound dressing during EBD procedures appeared to enhance esophageal wound healing, delaying the stricture formation process ( Fig. 3 ).

**Fig. 3:**
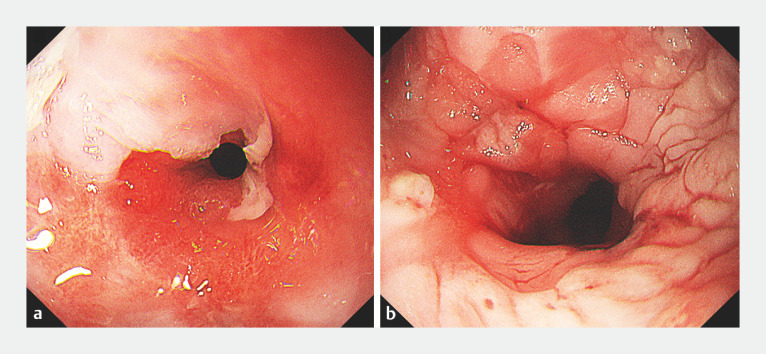
Gradual improvement of the stricture following the use of the self-assembling peptide hydrogel. **a** Before application. **b** After introduction of PuraStat.

In conclusion, our experience indicates that PuraStat may contribute to the prevention of re-stenosis after EBD for refractory postoperative stricture. PuraStat is suggested to be a valuable and safe option for managing not only bleeding prevention but also postoperative stricture.

Endoscopy_UCTN_Code_TTT_1AO_2AD

## Notes

Endoscopy E-Videos https://eref.thieme.de/e-videos
*E-Videos* is an open access online section of the journal *Endoscopy* , reporting on interesting cases and new techniques in gastroenterological endoscopy. All papers include a high-quality video and are published with a Creative Commons CC-BY license. Endoscopy E-Videos qualify for HINARI discounts and waivers and eligibility is automatically checked during the submission process. We grant 100% waivers to articles whose corresponding authors are based in Group A countries and 50% waivers to those who are based in Group B countries as classified by Research4Life (see: https://www.research4life.org/access/eligibility/ ). This section has its own submission website at https://mc.manuscriptcentral.com/e-videos .
